# Identifying an initial set of core components for perinatal cannabis use harm reduction counseling: An application of the Consensus on Relevant Elements (CORE) process

**DOI:** 10.3389/adar.2026.15935

**Published:** 2026-04-13

**Authors:** Ariana M. Albanese, E. Ruby Cramer, Hannah E. Frank, Brooke G. Rogers

**Affiliations:** 1 Department of Psychiatry and Human Behavior, The Warren Alpert Medical School of Brown University, Providence, RI, United States; 2 Psychiatry, Boston University Chobanian and Avedisian School of Medicine, Boston, MA, United States; 3 Psychiatry, Boston Medical Center, Boston, MA, United States

**Keywords:** breastfeeding, cannabis, core components, harm reduction, pregnancy

## Abstract

Cannabis use during pregnancy or lactation is a key topic in healthcare today. It is recommended that patients abstain from perinatal use and healthcare providers are encouraged to discuss use with patients. However, some perinatal individuals continue to use even once educated about the risks. For them, a nuanced approach to counseling that goes beyond warnings of risks is required to promote health and maintain trust. Harm reduction, which views any reduction in harm as a clinical success, is the best evidence-based practice for these patients. However, the essential elements (core components) of this approach have not yet been identified. This limits the approach’s readiness for wider implementation, as it is critical that essential elements are maintained during intervention adaptation and scaling. We used the Consensus on Relevant Elements process to generate an initial list of core components for harm reduction counseling of cannabis using-pregnant and breast-feeding patients. We identified five core components within two domains. Within the domain of provider training, the provider 1) is skilled at facilitating conversation, and 2) is educated about cannabis use. Within the domain of patient-facing work, the provider 3) educates patients about cannabis safety and recommendations, 4) screens for cannabis, and 5) provides a brief harm reduction intervention. Future work to achieve a broader consensus of the core components of this approach should be undertaken to confirm these findings. This work serves as an important first step towards equipping healthcare providers to promote health for perinatal cannabis-using patients.

## Introduction

Perinatal cannabis use, defined as cannabis use during pregnancy or lactation, is an important topic in healthcare today. Prenatal use is associated with adverse health effects [1], and tetrahydrocannabinol (THC) can be transferred to the infant via breastfeeding [2]. Thus, abstention is recommended and healthcare providers are encouraged to discuss use with their patients [3].

However, in an era of rapid legalization, rates of perinatal cannabis use are increasing [4], as is the perception of safety [5]. Further, some patients continue using despite knowledge of harms – either because they perceive benefits of use [6, 7] and/or have difficulty discontinuing use. For such patients, a nuanced approach to counseling that goes beyond warning of risks is needed. Critically, research on motivational enhancement treatment for patients with substance use disorders suggests that an abstinence-only approach is unlikely to promote health in such cases, as constraining an individual’s choice may lead them to be more firmly set in their behaviors [8–10]. Harm reduction offers an alternative approach. Within a harm reduction framework, any reduction in harm related to use of a substance is viewed a clinical success [11]. Harm reduction is thus the best available evidence-based practice for patients who are unable to completely cease use or remain abstinent.

We conceptualize a healthcare provider taking a harm reduction approach to the counseling around perinatal cannabis use as involving two aspects. The first is *what* is said, namely the provision of information on concrete behavior changes that lessen harm associated with use of a substance, such as the Fischer guidelines for lower risk cannabis use [12–14]. Equally important, is *how* things are said, meaning that providers embody key principles of harm reduction in healthcare settings such as humanism, pragmatism, and respect for autonomy [15].

In addition to being a good approach for individuals unable to abstain generally (from any substance) harm reduction is well-suited to perinatal cannabis use specifically. First, harm reduction approaches are included in clinical guidelines to treat other perinatal substance use disorders like opioid use disorder [16]. Also, there have been calls for research to integrate harm reduction into the treatment of cannabis use [17]. Additionally, perinatal patients are already employing strategies to reduce risk stemming from cannabis use [18], and report a desire for harm reduction education such as information about the safety of various modes of consumption [19]. Thus, there is both justification and demand for broader implementation of a harm reduction approach for perinatal cannabis use in clinical care.

However, before harm reduction for perinatal cannabis use can be more widely practiced, its essential elements (core components) must be identified. These core components can be thought of as “active ingredients” which, when reproduced with fidelity, causally enable the intervention to be effective [20]. Consideration of core components enables effective replication of interventions [21]. Additionally, efforts to spread an evidence-based practice often involve adapting it for new end-users and implementers. Identifying core components can foster mindful adaptation that maintains intervention fidelity [20]. To answer the need to identify core components of harm reduction for perinatal cannabis use we used to be Consensus on Relevant Elements (CORE) process [22] to generate an initial list of core components for this evidence-based practice.

## Materials and methods

A multiphase core component identification process took place with an expert panel and moderators. The expert panel consisted of three members with content expertise relevant to the intervention: implementation science (third author), behavioral substance use treatment including harm reduction counseling (fourth author), and perinatal health (first author). The two moderators (first and second authors) both had experience with facilitating group discussions and focus groups, including one moderator’s involvement in a modified Delphi process [23]. Of note, one moderator played a dual role as the panel expert on perinatal health. The overarching goal of the process was to identify what core components are required when taking a harm reduction approach to perinatal cannabis use counseling.

**FIGURE 1:**
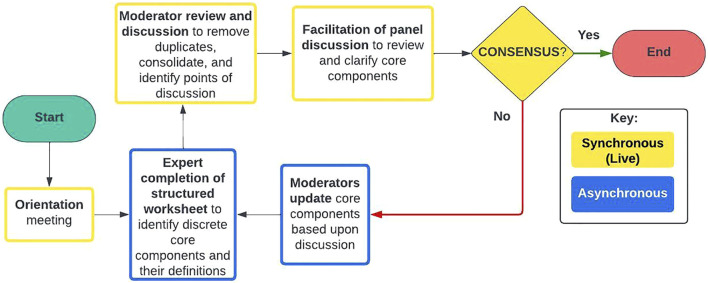
Flow diagram of the Consensus on Relevant Elements (CORE) Process. Note: image adapted from one created by Dr. Greg Rosen and used with his permission.

Our process involved an alternating sequence of asynchronous expert completion of worksheets which guided them through generating and refining core components and synchronous live meetings which featured moderated discussions of the completed worksheets. Moderators would review these worksheets ahead of discussions to prepare an agenda of key discussion points for each live meeting. This process is intended to be cyclical and repeated until consensus is reached. Our process involved two cycles of worksheet completion and discussion. One major development during the discussion of the first completed worksheet was clarifying the level of detail required in the description of a core component. The team realized that some of the descriptions were so detailed they were better classed as describing intervention content (the specifics of each intervention element) rather than describing core components (a higher-level description). Once this understanding was reached, the process of reaching consensus moved quickly. Our process is outlined in a flow diagram in Figure 1 and the steps of this process are described in detail in Table 1. The worksheets completed by the experts are also included as Supplementary Material.

**TABLE 1:** Steps of the core components analysis guided by the CORE process.

Step	Process
1	Initial drafting of core components by expert panel members
	a) The expert panel attended a meeting in which they were oriented to the intervention and the CORE process b) Following the meeting, worksheet A (Supplementary Material 1) was distributed to expert panel members as well as a copy of an educational pamphlet created to support providers in taking this approach [24]. Experts were instructed to independently complete worksheet A (an initial draft of core components) based on their review of the pamphlet and their own expert knowledge
2	Moderator review and consolidation of initial draft of core components
	a) Worksheets were returned to the moderators who reviewed the initial 23 suggestions generated in step 1 b) Clear duplicates were removed and the remaining suggested core components were reorganized thematically into four main components (each with multiple subparts). These were: 1) providers deliver key messages for patient education about perinatal cannabis, 2) providers have an up-to-date education on perinatal cannabis, 3) once cannabis use is identified, providers know what steps to take in a conversation with the patient to support them in reducing harm, 4) once cannabis use is identified, providers have the skills to facilitate patient emotional safety and promote trust using trauma-informed practice c) Moderator review and discussion generated several points for discussion with the expert panel. These included: 1) whether the core component of education for providers should be divided into the education providers must be able to give to the patient, vs. education that they themselves should have (and may or may not need to give to the patient, depending on the patient’s particular case and counseling needs), 2) whether reporting requirements to social services should be considered a key piece of education given to patients, 3) whether education on concrete behavioral change strategies that comprise lower risk cannabis use should be a necessary aspect of the provider education core component d) Moderators sent out this reorganized list (Supplementary Material 2) and discussion questions to the expert panel for review ahead of the facilitated discussion conducted in step three
3	Moderators’ facilitation of an expert panel meeting to discuss and clarify the suggestions of core components and their definitions
	a) A major focus of the expert panel discussion was what level of detail is required in the definition of the core component. The point was raised that much of the content in the core component “definition” column of worksheet A was highly specific and seemed to be describing the content of the intervention (the specific materials, activities, or strategies used to deliver the intervention) rather than a core component (the essential elements that are critical for achieving the intended outcomes). The decision was made to preserve the detail currently provided in the “definition” column as an “intervention content” column for future use, but to separate this out from the definition of the core component (Supplementary Material 3) b) As a result of this decision, most of the discussion questions raised in step two were rendered moot in the context of a discussion of core components as they were deemed to relate more to intervention content. However, related to core components, it was decided that education should be divided out into education for providers to give to all patients versus those that provider should have and may or may not relay to the patient depending on each patient’s needs
4	Moderators draft updated list of core components based upon the discussion in step three
	a) An updated list of core components was created by the moderators following the expert panel meeting in the preceding step. This was formatted into worksheet B (Supplementary Material 4). This new worksheet asked how the description could be improved, whether components should be merged or split, and for suggestions of short “codes” that can be used in reference to the components. Several items were highlighted in the worksheet for experts’ specific consideration b) Of note, as part of this step, the core components were organized into two domains (provider training and patient-facing work)
5	Experts provide revisions to core components list
	a) Worksheet B was sent to the expert panel to complete
6	Moderator review of suggested core component revisions
	a) Worksheets were returned to the moderators who reviewed suggested revisions and consolidated the revised components (Supplementary Material 5). Moderators incorporated revisions that were consistent across the panel and generated points for expert panel discussion related to suggestions which were not clearly consistent. These included: 1) whether to add a core component related to screening for cannabis use, 2) whether to provide further detail about the brief intervention core component b) Overall, feedback during this step did not suggest new core components (other than adding screening for cannabis use as a core component). Rather, feedback focused on workshopping phrasing of the already drafted components c) Moderators sent out this consolidated revised list and discussion questions to the expert panel for review ahead of the facilitated discussion conducted in step seven
7	Moderators’ facilitation of an expert panel meeting to discuss and clarify revisions to the core components
	a) Moderators facilitated a second discussion in which the revised consolidated core components list was reviewed. Stemming from this conversation, it was decided that a screening core component should be added and that further detail about the brief intervention was not necessary as further detail would fall into describing intervention content b) Given that expert suggestions were fairly minor and focused around phrasing of already agreed upon components, it was decided that another round of worksheet completion and discussion was not necessary. The moderators incorporated the expert suggestions stemming from this discussion and sent a final list for expert final approval via email. This resulted in the final list of core components (see Table 2)

## Results

Using the CORE process, which involved an expert panel and moderated discussion, we generated an initial list of five core components spread across two domains.

The first of these domains is provider training. Core components that fall into this domain are related to competencies that a provider should achieve (through training and mentorship) outside the context of, and ideally before arriving at, the clinical encounter. The first core component within this domain is that the provider is skilled at facilitating conversation. This means they understand how to be trauma-informed in a patient conversation (empower patients to make informed decisions, practice reflexivity, and incorporate an awareness of power differentials into their process of asking the patient questions). The second core component within this domain is provider education about cannabis use. This means providers have an up-to-date knowledge of the safety data and recommendations regarding cannabis use during pregnancy and breast-feeding. They should also possess a basic understanding of cannabis including knowledge of topics such as cannabinoids, modes of consumption, and behavior change strategies to reduce harm stemming from use.

**FIGURE 2:**
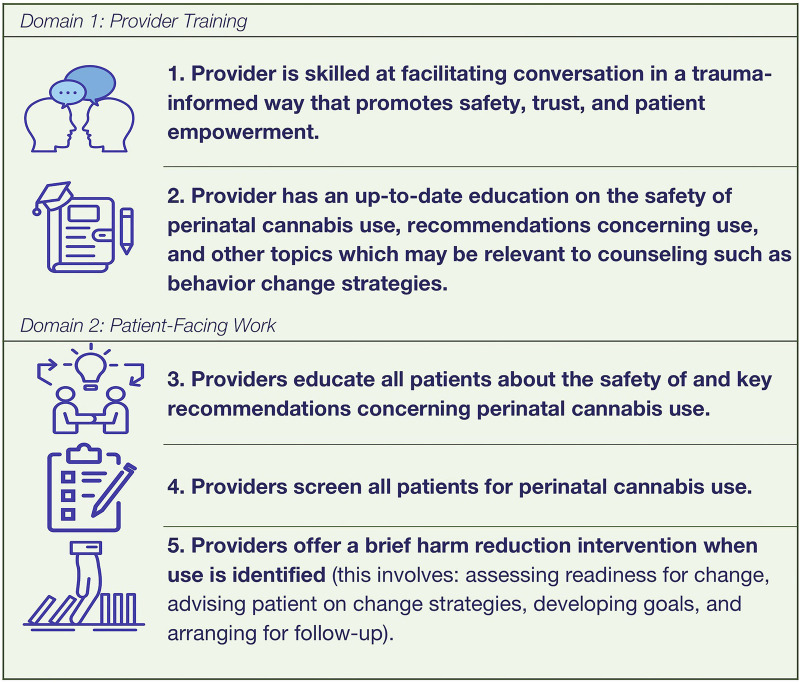
The identified core components of a harm reduction counseling intervention for perinatal cannabis use.

The second of these domains is patient facing work. This domain describes activities that the provider should perform in the context of the clinical encounter with the patient. The first core component in this domain is educating patients about safety recommendations concerning perinatal cannabis. This means that providers deliver key educational messages about the safety of use and that use is not recommended. This is done as soon as patients are contemplating pregnancy and should be done universally (regardless of whether the patient has reported use). The second core component is screening for perinatal cannabis use either verbally or using written measures. This should also be performed for all patients. The last core component is that providers conduct a brief harm reduction intervention. This is done as soon as cannabis use is identified and follows Substance Abuse and Mental Health Services Administration (SAMHSA) recommendations (educating about a effects, providing advice on change, assessing readiness for change, negotiating goals and strategies, and arranging follow-up) [25]. Figure 2 presents our final list of components. Detailed definitions of these core components can be found in Table 2.

**TABLE 2:** Core components of a harm reduction approach to counseling about perinatal cannabis use.

Title	Definition
*Domain 1: Provider Training*	
Provider is skilled at facilitating conversation	Providers are skilled in facilitating trauma-informed conversations that promote patient emotional safety and trust. These skills include the ability to: empower patients to make informed decisions, practice reflexivity, and ask questions in a trauma- informed way
Provider is educated about cannabis use	Providers maintain up-to-date education on perinatal cannabis: This education includes the core topics of safety during pregnancy and breastfeeding/chestfeeding, recommendations about perinatal use, limitations of safety data, as well as other topics which may be relevant to counseling. These other topics include: a basic understanding of primary cannabinoids (THC and CBD), the modes by which cannabis may be consumed, factors that can affect cannabis’ impact, a brief historical context to explain the legality and epidemiology of use, relevant language and terminologies for cannabis use, behavior change strategies to reduce harm stemming from use, knowledge of pregnancy- safe options, and resources to address symptoms patients may currently be treating using cannabis
*Domain 2: Patient-Facing Work*	
Educate patients about the safety of and recommendations concerning perinatal cannabis use	As soon as patients are contemplating pregnancy, providers deliver key educational messages about perinatal cannabis use (e.g., current understandings of the safety of perinatal use and that use is not recommended during pregnancy/lactation) as well as the limitations of existing data. This can be done when having discussions about other behaviors (i.e., prenatal vitamins, consuming alcohol, exercising, etc.)
Screen for perinatal cannabis use	Verbally or using a written measure (such as TAPS I and TAPS II, DAST-10, and the NIDA quick screen), providers and patients engage in a brief assessment to screen for cannabis use for all patients. This assessment includes distinguishing whether use is recreational or medicinal, if use is endorsed
Provide a brief harm reduction intervention	As soon as cannabis use is identified, providers implement a brief intervention for cannabis use. They follow the substance Abuse and mental health services Administration (SAMHSA) recommendations [25] for brief intervention on cannabis use which includes educating about effects, providing advice on change, assessing readiness for change, negotiating goals and strategies, and arranging follow-ups. If the patient is ready to cease use, goals and strategies for change will focus on cessation, if that patient is not ready to cease use, goals and strategies for change will focus on reducing the harm stemming from continued use (this may involve a reduction in use or other evidence-based behavioral changes which reduce health harms)

## Discussion

Despite the data demonstrating negative parent and child health outcomes associated with perinatal cannabis use [1], cannabis use in pregnant and lactating individuals is on the rise [4]. In the context of rapid legalization, cannabis is becoming more accessible and accepted and safety perceptions around its use are changing accordingly [5]. For a subset of perinatal individuals, the perceived benefits of use outweigh the potential health risks–leading them to continue use even after being educated about the risks [6, 7]. A harm reduction approach to counseling about perinatal cannabis use is the best available evidence-based practice to promote health for patients who choose to continue to use cannabis despite knowledge of potential harms. However, this practice has yet to be widely implemented despite both theoretical justification and patient demand. A crucial step that must precede wider adoption is identifying the core components of harm reduction for perinatal cannabis use. This will ensure that these components are maintained when the intervention is adapted and scaled. We sought to answer this need by using the CORE process to generate an initial list of core components of harm reduction for perinatal cannabis use. We ultimately identified five core components within two domains. Within the domain of provider training, the provider 1) is skilled at facilitating conversation, and 2) is educated about cannabis use. Within the domain of patient-facing work, the provider 3) educates patients about the safety of and recommendations concerning PCU, 4) screens for PCU, and 5) provides a brief harm reduction intervention.

The identification of the core components of harm reduction for perinatal cannabis use underscored that harm reduction constitutes a not merely a set of concrete instructions, but requires a provider’s philosophical shift away from traditional paternalistic understandings of the patient – provider relationship. Traditionally, the provider occupied the role of giving directions to promote health and the patient occupied the role of following them. Harm reduction requires that providers *partner with* their patients, respecting patient autonomy to make their own decisions and contending with feelings of moral ambiguity surrounding patients making healthcare decisions that a provider might not agree with [15]. This philosophical shift is encapsulated in multiple of the core components. First, the facilitation skill core component centers around being able to conduct a trauma-informed conversation which is an approach that explicitly seeks to return power to the patient through emphasizing their autonomy and seeking permission to ask questions [26]. Second, the performance of the brief harm reduction intervention is based on an assessment of readiness for change. If the patient is not in a place where they are able or willing to change their behaviors, behavior changes should not be sought. It may be challenging for providers to adopt this shift given the aforementioned concerns about enabling harm, especially in a perinatal population, where the parents’ decisions impact both the patient and their infant. Thus, special attention will need to be paid to developing implementation strategies that support use of harm reduction. One strategy which may be helpful is disseminating evidence about the positive patient outcomes associated with a harm reduction approach. In addition to the already cited work demonstrating the ineffectiveness of abstinence only approaches for individuals who are unable or unwilling to stop use [8–10], adopting a shared decision-making model has also been shown to be beneficial for substance using individuals. Shared decision-making, in which patients have “freedom of choice” surrounding health behavior decisions, is both preferred by substance-using patients [27] and is linked to positive health outcomes like greater patient utilization of care [28]. Additionally, strategies focused on de-implementation or discontinuation of the traditional paternalistic model of care will also likely be a key pairing with strategies promoting implementation of harm reduction.

### Strengths and limitations

This study has several strengths including the application of a formal methodological process for generating an initial list of core components. Additionally, as part of this process, the expert panel brought a range of relevant expertise. Importantly, these experts brought both academic and clinical expertise via their practices as licensed clinical psychologists. More specifically, one of the experts (fourth author) has years of experience employing harm reduction practices with patients. Another expert (first author) has years of experience working with the perinatal population. This clinical experience, alongside academic knowledge, enhanced the perspective that each expert brought to the core components identification process. Finally, despite not explicitly featuring patients/potential recipients of this approach, all three expert panel members have lived experience receiving perinatal care, strengthening the panel with their personal insights and perspectives as patients.

Despite its strengths, the study also has several limitations. First, the authors of the CORE process note that the involvement of intervention developers in drafting initial core components is important. Unfortunately, the team who developed a pamphlet describing this approach [24] was unable to be involved in this process due to time constraints and competing responsibilities. However, they were supportive and encouraged our use of their materials. Additionally, as mentioned, one expert panel member (first author) participated as both an expert panel member and moderator. This dual role may have affected their intended neutrality as a moderator and ability to share as an expert, as their attention was split between sharing expertise and moderating the discussion. Also, our expert panel did not feature a prenatal healthcare provider such as a physician or midwife, though it did feature a perinatal behavioral health provider. Additionally, the example application of the CORE process presented by Kalver and colleagues (2021) mentioned that several intervention materials (manual, protocol) were analyzed by experts in the process of identifying core components. Given the nature of our intervention, which is not a formally protocolized and tested intervention, we did not have as many materials to review. Lastly, our expert panel was small, which may limit the generalizability of these core components or their ability to fully capture patient complexity. We do note that our panel was comparably sized to that which was used by Kalver and colleagues in their initial application of the CORE process [22]. However, this necessitates further consensus-building work to ensure component identification is generalizable and complete (discussed in “Future Directions”).

### Future directions

As discussed by Kalver and colleagues (2021) in their description of the CORE process, the identification of core components is often a multistep endeavor. The CORE process provides a systematic series of steps for generating *an initial list* of core components. Especially given the size of our expert panel, it is important to perform broader consensus work, such as a Delphi study, to confirm these findings. Additionally, subsequent testing of the core components of harm reduction for perinatal cannabis use enables refinement of the list over time. Thus, future research should first seek confirmation of these findings, then employ methods such as those described by Haynes and colleagues to test these core components in clinical studies and further refine them [29]. Additionally, as discussed, future work should develop and test implementation strategies to support uptake and sustained practice of harm reduction counseling for perinatal cannabis use. Lastly, but importantly, the field currently lacks conclusive research on the health impacts of perinatal cannabis use. Given that providing education on safety is a core component of this intervention, future work should better clarify health risks so that provider recommendations can be more conclusively evidence-based.

## Conclusion

This work meaningfully adds to the literature on health promotion for perinatal cannabis-using patients as it begins to define the necessary elements which must be present to effectively enact harm reduction counseling for perinatal cannabis use. By beginning to identify the concrete elements that must be included in harm reduction for this population, calls for increased implementation of a harm reduction approach to perinatal cannabis use [17, 19] can begin to be addressed with practical clinical guidance.

## Data Availability

The original contributions presented in the study are included in the article/Supplementary Material, further inquiries can be directed to the corresponding author.
