# Supplementary material for: Identifying an initial set of core components for perinatal cannabis use harm reduction counseling: An application of the Consensus on Relevant Elements (CORE) process
Source: Adv Drug Alcohol Res. 2026 Apr 13;6:15935. doi: 10.3389/adar.2026.15935 (PMC13111171; doi:10.3389/adar.2026.15935)
Supplement: Supplementary file 1 [file Supplementaryfile1.docx]

**Supplementary File 1**

**WORKSHEET A**

**Expert panel members’ individually brainstormed descriptions of the innovation’s core components. Note: the innovation is operationalized as taking a harm reduction approach to the education and counseling about perinatal cannabis use (encapsulated by the Perinatal Services BC “Cannabis Use During Pregnancy & Lactation”, practice resource, “PSBC Resource”, and enhanced by the expert panel’s content area expertise)**

*To be completed by expert panel members prior to the facilitated discussion on what the core components of the innovation are*

Information requested of discussion participants:

- Please fill in the table below on what you consider to be core components of a harm reduction approach to education and counseling around perinatal cannabis use.
- To identify these components:
  - look at the PSBC Resource, and breakdown its “anatomy” (what are the elements it is representing – e.g., foci of education)
  - bring your own expert understanding to bear (for EXPERT NAME – what elements must be included in order for the spirit and practice of harm reduction to be captured; for EXPERT NAME – what are key elements that must be included for effective implementation of a discussion guide in health care context)
- In filling out the table, please feel free to use your own wording to designate/describe/define the components or wording used in the innovations source materials (i.e., the PSBC Resource).
- Please add rows to the table as needed.

| **Core component** | **Definition (who, what, where, when, why)** |
| --- | --- |
|  |  |
|  |  |
|  |  |
|  |  |
|  |  |
|  |  |
|  |  |

*Thank you!*
