# Supplementary material for: Identifying an initial set of core components for perinatal cannabis use harm reduction counseling: An application of the Consensus on Relevant Elements (CORE) process
Source: Adv Drug Alcohol Res. 2026 Apr 13;6:15935. doi: 10.3389/adar.2026.15935 (PMC13111171; doi:10.3389/adar.2026.15935)
Supplement: Supplementary file 2 [file Supplementaryfile5.docx]

**Supplementary File 5**

**WORKSHEET B**

|  | **What should this component be called (give it a short “code” that we can use to refer to it)** |
| --- | --- |
| Domain 1: Provider Training | |
| 1. **Consolidated version:** Providers are skilled in trauma-informed conversation facilitation that promotes patient emotional safety and trust. These skills include the ability to empower patients, practice reflexivity, and ask questions in a trauma informed way. | **Provider conversation facilitation skills** |
| 2. **Consolidated version:** Providers maintain up-to-date education on perinatal cannabis**.** This education includes the core topics of safety during pregnancy and breastfeeding, recommendations about perinatal use, limitations of safety data, as well as other topics which may be relevant to counseling. These other topics include: a basic understanding of THC and CBD, the modes by which cannabis may be consumed, factors that can affect cannabis’ impact, the epidemiology of cannabis use including legality and its effects on use, behavior change strategies to reduce harm stemming from use, knowledge of pregnancy safe options and resources to address symptoms patients may currently be treating using cannabis. | **Provider education about cannabis** |
| Domain 2: Patient-facing work | |
| 3. **Consolidated version**: As soon as patients are contemplating pregnancy, providers deliver key educational messages about perinatal cannabis use (e.g., current understandings of the safety of perinatal use and that use is not recommended during pregnancy/lactation) as well as the limitations of existing data. | **Consolidated version:**  **Patient education about the safety of and recommendations concerning perinatal cannabis use** |
| 4. **Consolidated version:**  As soon as cannabis use is identified, providers implement a brief intervention for cannabis use. They follow the Substance Abuse and Mental Health Services Administration (SAMHSA) recommendations for brief intervention on cannabis use and include educating about effects, providing advice on change, assessing readiness for change, negotiating goals and strategies, and arranging follow-ups. | **Consolidated version:**  Brief intervention |
