# Supplementary material for: Identifying an initial set of core components for perinatal cannabis use harm reduction counseling: An application of the Consensus on Relevant Elements (CORE) process
Source: Adv Drug Alcohol Res. 2026 Apr 13;6:15935. doi: 10.3389/adar.2026.15935 (PMC13111171; doi:10.3389/adar.2026.15935)
Supplement: Supplementary file 3 [file Supplementaryfile3.docx]

**Supplementary File 3**

**WORKSHEET A**

**Expert panel members’ individually brainstormed descriptions of the innovation’s core components. Note: the innovation is operationalized as taking a harm reduction approach to the education and counseling about perinatal cannabis use (encapsulated by the Perinatal Services BC “Cannabis Use During Pregnancy & Lactation”, practice resource, “PSBC Resource”, and enhanced by the expert panel’s content area expertise)**

*To be completed by expert panel members prior to the facilitated discussion on what the core components of the site innovation are*

Information requested of discussion participants:

- Please fill in the table below on what you consider to be core components of a harm reduction approach to education and counseling around perinatal cannabis use.
- To identify these components:
  - look at the PSBC Resource, and breakdown its “anatomy” (what are the elements it is representing – e.g., foci of education)
  - bring your own expert understanding to bear (for PANEL MEMBER NAME – what elements must be included in order for the spirit and practice of harm reduction to be captured; for PANEL MEMBER NAME – what are key elements that must be included for effective implementation of a discussion guide in health care context)
- In filling out the table, please feel free to use your own wording to designate/describe/define the components or wording used in the innovations source materials (i.e., the PSBC Resource).
- Please add rows to the table as needed.

| **Core component** | **Definition (who, what, where, when, why)** | **Intervention content** |
| --- | --- | --- |
| **Providers Have Skills in conversation facilitation** | Once cannabis use is identified, providers have the skills to facilitate patient emotional safety and promote trust using a trauma-informed practice. | *On the context vis-a-vis providers:*   - Recognize providers may be unfamiliar with this approach and it may not be a skill they are fluent in (yet) - Recognize this may feel morally challenging when in opposition with their work towards an optimal fetal environment and empathize with providers here - Recognize providers may have facilitated similar conversations in regards to discussing risk of use (ie., psychiatric medications) while pregnant   *On empowering patients:*   - Align with the patient. Be on their side. - Collaboratively engage in discussion (back and forth) to problem solve   - Give an example? - Offer an understanding for the rationale behind cannabis use - Normalize cannabis use & the discussion around cannabis use - Understand/acknowledge the potential for trauma reactions - Identify client strengths   - Goals   - Coping skills - Check-in with patients throughout conversation - Acknowledge the potential for trauma reactions - Emphasize the client’s autonomy during the conversation   - What do they think is best for their own bodies and baby?     - Be genuinely curious as to why this is best for them - Use gender inclusive language when it serves patient (ie, chestfeeding)   *On reflexivity:*   - Providers should be reflexive of positions of power and privilege - Assess and consider cultural norms and context - Engage in reflective listening - Take a non-judgemental stance   *On asking questions:*   - Use open-ended questions - Limit the number of questions, especially when all in a row - to improve power dynamic - Provide rationale for questions   - Offer to support them through pregnancy as a provider, regardless of their choices. - Keep conversation open |
| **Providers are Educated on Perinatal Cannabis** | Providers are educated on primary prevention messages for all patients (safety during pregnancy and breastfeeding, recommendations about perinatal use), providers are educated on additional information about cannabis which they can draw on *as counseling needs dictate [secondary and tertiary prevention].* | Providers have to have a broad scope of education from which to pull for educating patients on a case-by-case basis, [in addition to the core key messages, item #3]  Recommendation concerning perinatal use;   - It is recommended to discontinue perinatal use, reduce if not possible to stop, and/or take other behavioral approaches to reduce risk   Safety Data re: Impacts on baby   - Providers are aware of the potential risks to birth outcomes such as low birthweight, small gestational age, stillbirth, and preterm birth - Providers are aware of the potential neurological impacts on the baby such as memory, attention, mood, sleep, decision making, hyperactivity, and future substance use   Safety data re: Impacts of using while chest/breastfeeding   - reduced milk production - transfer of chemicals via milk - potential of these chemicals to impact neurological development   Limitations of safety data   - Providers are aware that given cannabis is commonly used in combination with other drugs, knowledge of independent effects is limited   - confounding exposures - Socioeconomic conditions that are compounding factors   - poverty, malnutrition, education, household income, supplement use by pregnant folks, intimate partner violence, - Studies are subject to reporting and recall bias   - rely on self-reported habits such as frequency, timing, amount, - Potency of cannabis (ie., cbd/thc %) has risen with time and legality - Pregnant folks are a vulnerable population and research on them is protected - Lack of knowledge and research on chest/breastfeeding outcomes   Knowledge of cannabis consumption   - Providers are aware that THC and CBD are found in cannabis and understand their basic effects on the human body - The multiple modes by which to consume cannabis. They are familiar with these modes and how quickly effects are felt by the user. Not currently included in PSBC resource: providers are also aware of the relative risks associated with different modes (e.g. smoking is not for long health, edibles can be harder to dose safely given delayed effects)   - Smoking, vaping, cannabis oil, edibles, tinctures, dabbing or shattering - Providers are aware of other factors that can impact the effect felt by cannabis (dosage, previous experience with the substance).   Epidemiology of cannabis use   - Legality (broadly and state/locally specific) and awareness that legalization is increasing.   - able to communicate potential legal consequences   - reporting requirements of providers / DCYF involvement requirements - Awareness that legalization is likely impacting attitudes/understandings of cannabis   - Perception of safety - Awareness that legalization is likely impacting access to cannabis   - Costing, trustworthiness, - Prevalence of use in the population   - Prevalence of use among pregnant/breastfeeding folks (commonality of cannabis use during pregnancy)   Knowledge of behavior change strategies to reduce harm stemming from use   - Academy of breastfeeding medicine guidance - Lower risk cannabis use guidelines - Behavioral change strategies   - stimulus control (reducing cues to use – not being around others using, removing paraphernalia, etc.)   - gradual reduction (over the course of a few weeks); generally reduce frequency   - finding a replacement behavior during the times it was used (e.g., drinking seltzer water, chewing gum)   - find new sources of support who do not use cannabis. offer connection to new moms groups that support healthy habits to collaboratively identify tailored harm reduction goals   - change form of use (eg, switch from smoking to edibles)   - reduce potency (ie., strain with less THC)   - acquire from a safe place (dispensary vs street)   Knowledge of pregnancy safe options & resources to address why patients might be using cannabis and/or to get them additional support as needed   - Pregnancy safe sleep aids, nausea medications, appetite stimulants, medications, etc. - Programs to support patients with situational stressors: economic challenges, housing instability, domestic violence support, food insecurity, etc. - Connections to specialized care - Mental health resources   Providers have access to updated information about the impact of perinatal cannabis use as new studies come out (know where to look, are easily able to check). |
| **Providers Deliver Key Patient Education** | As soon as patients are “contemplating pregnancy” and in a trauma-informed conversation style, providers pull from their education to effectively (meaning in a way the patient can understand) educate patients on key messages about cannabis use during pregnancy/lactation [primary prevention] and on limitations of existing safety data that informs recommendations. | - When: as soon as people are “contemplating pregnancy” - How: non-judgmental, trauma-informed, harm-reduction focused conversation style and approach [core component # 1] - What:   - Potential impacts of cannabis on pregnancy/fetal outcomes, the limitations of the data, the recommendations regarding use     - no known safe amount     - not recommended to improve conditions that may be experienced during pregnancy (nausea, vomiting, depression, anxiety)     - avoid smoking and/or exposure to second and third hand smoke     - may be associated with adverse outcomes on birth and neurological development     - may not be safe during breastfeeding – THC travels through breastmilk and can be present 30 days later     - recommended to avoid; if it cannot be avoided recommended to reduce or engage in other behavioral techniques to reduce health harms     - Legal risks/reporting requirements - Limitations of safety data (see details below) |
| Providers lead patients in a discussion of their cannabis use | Providers implement SAMHSA recommendations for brief intervention on cannabis use (educate about effects, provide advice on change, assess readiness, negotiate goals and strategies, arrange follow-up). | Providers can implement SAMHSA recommendations of how to briefly intervene when cannabis use is reported, doing so in a trauma-informed and harm-reduction style (as detailed in the next core component)   1. Provide education on health effects and potential legal consequences, 2. Inform clients of lower risk consumption practices [lower risk cannabis use guidelines] and provide advice on how to change behavior 3. Assess readiness for change, using motivational interviewing to assess pros and cons 4. In a pragmatic way [Hawk principles-pragmatic] negotiate goals and strategies for change    1. Understand their unique situation including discussion and consideration of unique socioeconomic and sociodemographic factors that can impact risk which the patient may not be able to control. Make sure usage patterns are understood e.g. frequency, quantity, method, concurrent substance use, others using around them. **Understand why they are using cannabis**. [Hawk principle-humanism]    2. Use this information in combination with lower risk cannabis use guidelines, Academy of breastfeeding medicine guidance, the understanding of why they use [are there other ways to address sxs currently addressed with cannabis?], and other **behavioral change strategies** (stimulus control [reducing cues to use – not being around others using, removing paraphernalia, etc.], reducing gradually over the course of a few weeks, finding a replacement behavior during the times it was used [e.g., drinking seltzer water, chewing gum] find new sources of support who do not use cannabis [offer connection to new moms groups that support healthy habits] to collaboratively identify tailored harm reduction goals],       1. collaboratively go back and forth to problem solve through asking open-ended questions. give **examples of things to ask** about such as: how much do you buy and at what interval (ie; 8gs/week). How much do you use upon consumption (ie., one joint every night)? 5. Arrange follow-up appointments to engage in continued discussion. “Celebrate positive incremental movement, do not penalize backwards movement but help patients understand ramifications [Hawk principles]” |

*Thank you!*
