# Supplementary material for: Identifying an initial set of core components for perinatal cannabis use harm reduction counseling: An application of the Consensus on Relevant Elements (CORE) process
Source: Adv Drug Alcohol Res. 2026 Apr 13;6:15935. doi: 10.3389/adar.2026.15935 (PMC13111171; doi:10.3389/adar.2026.15935)
Supplement: Supplementary file 5 [file Supplementaryfile4.docx]

**Supplementary File 4**

**WORKSHEET B**

Background:

- Below are descriptions of 4 potential core components of a harm reduction approach to medical provider education and counseling around perinatal cannabis use.
- These descriptions are based on the recent facilitated expert panel discussion, supported by wordings within the pre-discussion document (Worksheet A) completed by each panel member.
- Please excuse any inaccurately captured descriptions. If you see any in this document, we ask that you please suggest corrections in your answers below. Highlighted items seek your additional input. Also, if you have any feedback about the order of the components, please feel free to share that.

Instructions (Part 1 of 2):

- **For each of the 4 descriptions below, please briefly indicate:**

1. **How the description can be better worded**
2. **Whether the component should be further split into multiple components or merged with other components**
3. **What the component (or components, if suggesting a split into multiple components) being described should be called (i.e., a short “code” that can be used to refer to the component)**
4. **Whether the component(s) should be moved to be under a different domain**

|  | **How can this description be better worded?** | **Should this component be further split into multiple components? Merged with other components?** | **What should this component be called (give it a short “code” that we can use to refer to it)** |
| --- | --- | --- | --- |
| Domain 1: Provider Training | | | |
| 1. **Providers have skills** SHOULD THIS BE REFERRED TO AS “HAVE BEEN TRAINED IN?” **in conversation facilitation:** once cannabis use is identified, providers have the skills to facilitate patient emotional safety and promote trust using a trauma-informed practice. SHOULD MORE DETAIL BE PROVIDED HERE ABOUT WHAT CONSTITUTES A TRAUMA INFORMED PRACTICE/THE SPECIFIC SKILLS PROVIDERS NEED TO HAVE E.G. “THIS INCLUDES…”? |  |  |  |
| 2. **Providers are educated on perinatal cannabis:** providers are educated on primary prevention messages for all patients (safety during pregnancy and breastfeeding, recommendations about perinatal use), providers are educated on additional information about cannabis which they can draw on *as counseling needs dictate [secondary and tertiary prevention].* SHOULD MORE DETAIL BE PROVIDED HERE ABOUT THE SPECIFIC TOPICS WHICH PROVIDERS SHOULD BE EDUCATED ON E.G. “THIS INCLUDES”?  SHOULD WE CONSIDER ANOTHER CORE COMPONENT HERE ABOUT MAINTAINING EDUCATION (STAYING UP-TO-DATE ON NEW FINDINGS)? |  |  |  |
| Domain 2: Patient-facing work | | | |
| 3. **Providers deliver key patient education:** as soon as patients are “contemplating pregnancy” and in a trauma-informed conversation style, providers pull from their education to effectively (meaning in a way the patient can understand) educate patients on key messages about cannabis use during pregnancy/lactation [primary prevention] and on limitations of existing safety data that informs recommendations. SHOULD MORE DETAIL BE PROVIDED HERE ABOUT THE SPECIFIC TOPICS WHICH PROVIDERS SHOULD BE EDUCATING ON E.G. “THIS INCLUDES”? |  |  |  |
| 4. **Providers lead patients in a discussion of their cannabis use:** as soon as cannabis use is identified SHOULD THERE BE A DISCUSSION OF SCREENING AS A CORE COMPONENT OR A PART OF THIS COMPONENT, OR DO WE CONSIDER THIS HARM REDUCTION INTERVENTION TO TAKE PLACE AFTER USE IS IDENTIFIED? providers implement Substance Abuse and Mental Health Services Administration (SAMHSA) recommendations for brief intervention on cannabis use (educate about effects, provide advice on change, assess readiness, negotiate goals and strategies, arrange follow-up). SHOULD MORE DETAIL BE PROVIDED HERE ABOUT E.G. “THIS INCLUDES…”? |  |  |  |

Instructions (Part 2 of 2):

- **For each of the two domains below, please indicate:**

1. **Whether the domain should be further split into multiple domains or merged with other domains**
2. **How the name of the domain (or domains, if suggesting a split into multiple domains) can be improved**

|  | Should this domain be split? Merged with other domains? | How can the name of the domain (or domains, if suggesting a split) be improved? |
| --- | --- | --- |
| Domain 1: Provider Training |  |  |
| Domain 2: Patient-facing work |  |  |
